# Ectopic scrotum and penoscrotal transposition: Case report and literature review

**DOI:** 10.3389/fped.2023.1015384

**Published:** 2023-02-21

**Authors:** Haoran Huang, Xiangxia Liu, Zuoqing Li, Junjie Lin, Hai Yang, Zhe Xu

**Affiliations:** ^1^ Department of Pediatric Surgery, The First Affiliated Hospital, Sun Yat-sen University, Guangzhou, China; ^2^ Department of Plastic Surgery, University of Tennessee Health Science Center, Memphis, TN, United States; ^3^ Department of Pediatric Surgery, Meizhou People’s Hospital, Meizhou, China

**Keywords:** ectopic scrotum, rotation flap scrotoplasty, orchiopexy, anorectal malformation, VATER/VACTERL association

## Abstract

**Background:**

Ectopic scrotum (ES) is an extremely rare congenital scrotal malformation. Ectopic scrotum with VATER/VACTERL [vertebral defects (V), anal atresia or anorectal malformations (A), cardiac defects (C), tracheoesophageal fistula with or without esophageal atresia (TE), cardiac defects, renal malformations (R), and limb defects (L)] association is even rarer. There are no uniform guidelines for diagnosis and treatment.

**Clinical case:**

We described a 2-year-5-month-old boy who has ectopic scrotum and penoscrotal transposition and reviewed relevant literature in this report. We performed laparoscopy exploration, rotation flap scrotoplasty, and orchiopexy and achieved a great result during the postoperative follow-up.

**Conclusions:**

Combined with the previous literature, we made a summary to come up with a plan for the diagnosis and treatment of ectopic scrotum. Rotation flap scrotoplasty and orchiopexy are worthy of considering operative methods in treating ES. For penoscrotal transposition or VATER/VACTERL association, we can treat the diseases individually.

## Introduction

Ectopic scrotum (ES), commonly accompanied by other congenital malformations, is a kind of congenital scrotal anomaly. Mainly found in the inguinal, suprainguinal, infrainguinal, or perineal area, ES can occur anywhere from the inguinal canal and the perineum to the middle of the thigh (1–4). Depending on the relative position of the penis and scrotum, penoscrotal transposition can be classified into incomplete transposition and complete transposition. Incomplete transposition means that the penis is observed in the middle of the scrotum, while complete transposition refers to the penis located in the perineum below the scrotum. The acronym VATER/VACTERL association (OMIM #192350) is a synthesis of a series of congenital malformations, which refers to vertebral defects (V), anal atresia (A) or anorectal malformations (ARM), cardiac defects (C), tracheoesophageal fistula with or without esophageal atresia (TE), renal malformations (R), and limb defects (L). Generally, the diagnosis of VATER/VACTERL association requires at least three component features mentioned above (5–7). As one of the core features, ARM is often accompanied by genitourinary anomalies such as renal agenesis and hypospadias (8, 9). In addition, some patients diagnosed with VATER/VACTERL association also have other malformations besides the core features. Some of these patients should be diagnosed as other syndromes. For instance, patients with the presence of ear anomalies, choanal atresia, and developmental delay should be diagnosed with CHARGE syndrome (OMIM #214800) (10). However, the presence of ES with VATER/VACTERL association is extremely rare. We describe our treatments of a child who has ES and penoscrotal transposition. Interestingly, some clinical symptoms and signs of this child are similar to parts of the VATER/VACTERL association.

## Case description

**Figure 1:**
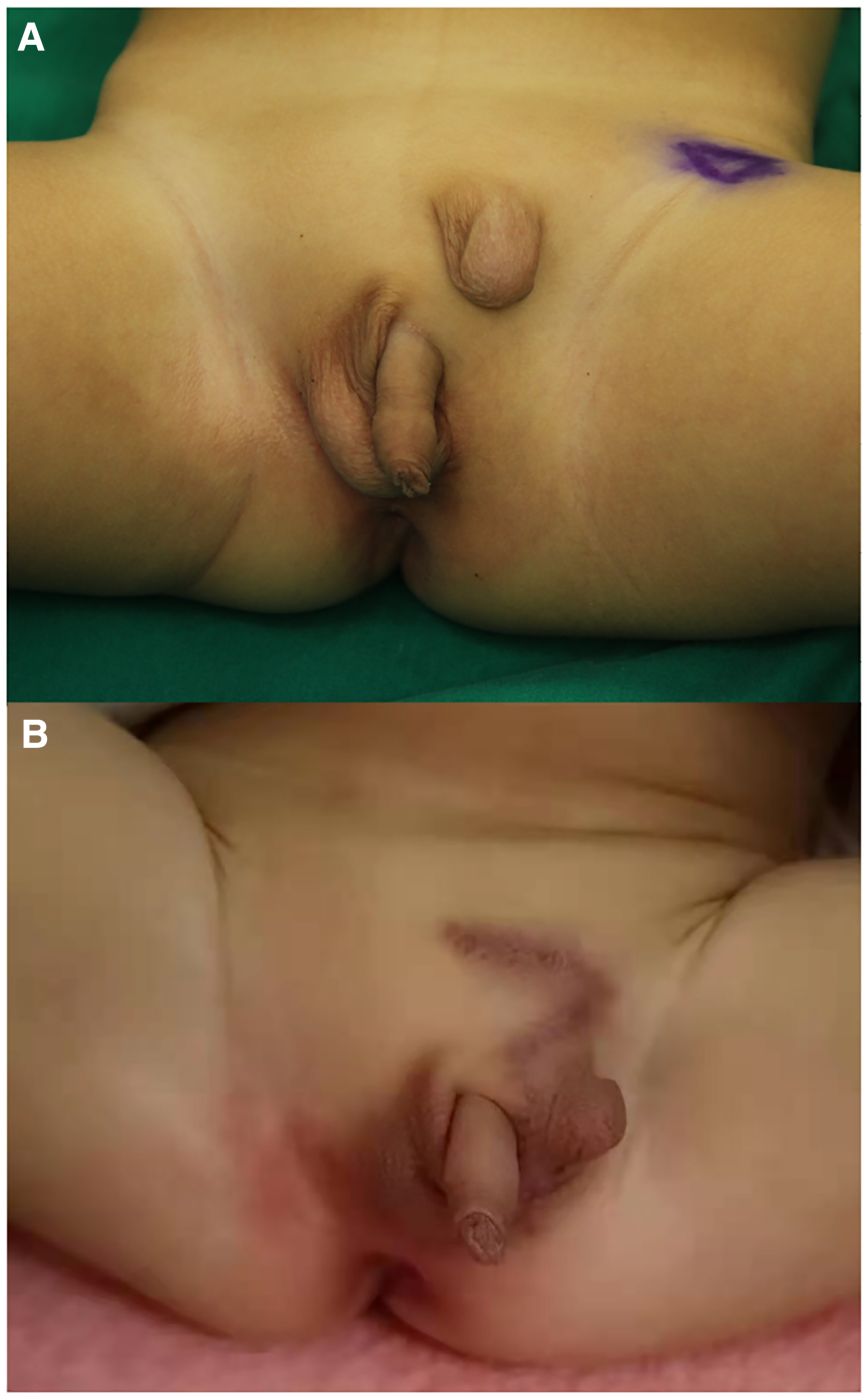
Preoperative and postoperative appearances of ES. (**A**) Preoperative. (**B**) Postoperative. ES, ectopic scrotum.

**Figure 2:**
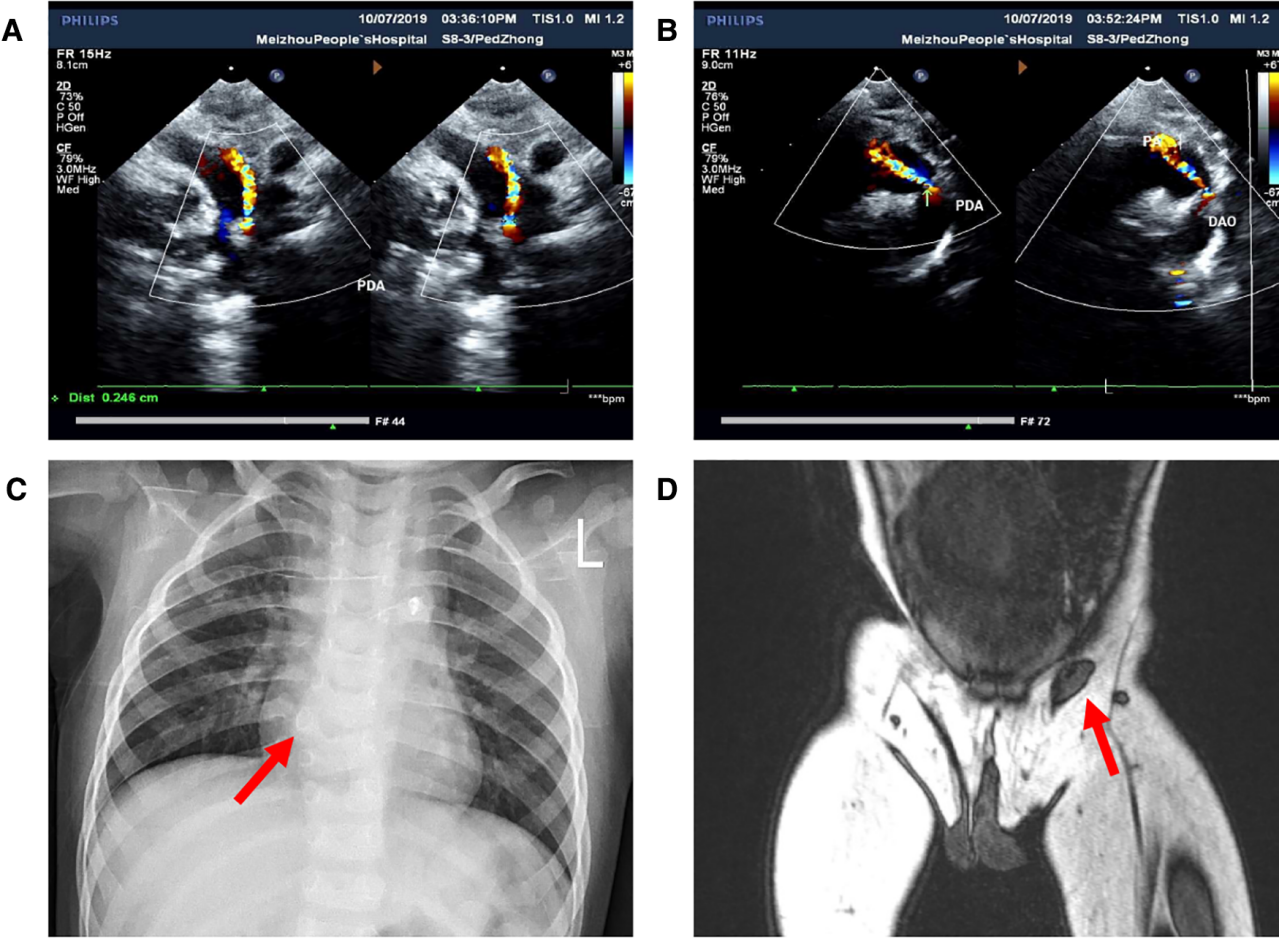
Images of preoperative auxiliary examination. (**A,B**) Patent ductus arteriosus detected by ultrasonic cardiogram; (**C**) hemivertebra deformity of the T8 vertebra detected by x-ray; (**D**) location of the left testis detected by MRI.

Born in Meizhou People's Hospital, a male newborn had ES, penoscrotal transposition, and ARM. Physical examination of this neonate revealed that there was no opening in the anus but a needle-like fistula in the perineum. The penis is observed in the middle of the right scrotum. Above the left groin, the ES was empty and small in size, while the position and size of the right scrotum with palpable testis inside is normal (Figure 1A). There was no history of inherited disorder in this child's family. The mother of the child did not take any drugs that might affect the development of the fetus during pregnancy and the delivery went well. During the initial hospitalization, we treated the ARM of the child by anal dilatation. At the same time, ultrasonic cardiogram showed patent foramen ovale and patent ductus arteriosus (PDA) (Figures 2A,B). Following up for more than 1 year, we found that the ductus arteriosus was still not closed. Thus, occlusion of patent ductus arteriosus was performed in the second hospitalization. Due to family circumstances, the child was treated in the First Affiliated Hospital of Sun Yat-sen University after 2 years old. Digital radiography showed hemivertebra deformity of the T8 vertebra (Figure 2C). The examinations of MRI, CT, and US suggested that the left testis was located in the left inguinal canal (Figure 2D). We did not find any obvious abnormalities in other pelvic organs and the urinary system.

**Figure 3:**
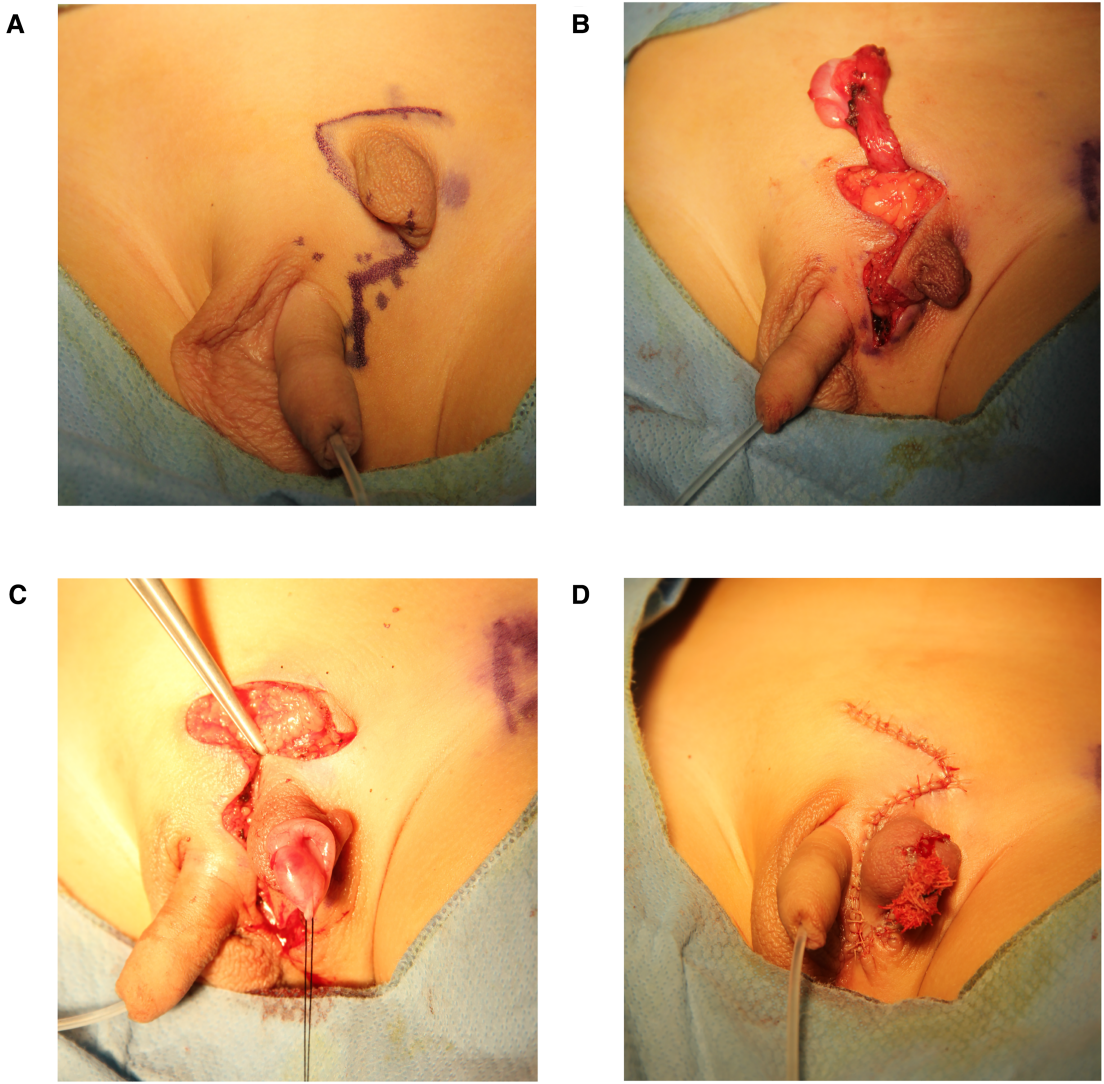
**(A-D)** Photographs of the surgery procedure.

This child underwent laparoscopy exploration, rotation flap scrotoplasty, and orchiopexy after consultation. First, under laparoscopic exploration, we found that the bilateral deep inguinal rings were closed. The left spermatic vessels and vas deferens entered the inguinal area through the left internal ring. Taking out the laparoscope, we started the second part of the surgery. To design the size and position of the skin flap, Doppler ultrasound was used to identify the cutaneous perforator vessels. The success of flap surgery depends largely on the size, distribution, and variation of perforator vessels (11). We cut and dissected the skin flap from the ES to the right scrotum using an inverse Z-shaped incision. It was important to protect the cutaneous perforator vessels of the flap. After exposing the inguinal canal and dissociating the spermatic cord in the left inguinal area, the left testis with epididymis was located at the internal ring. The ES was transferred to the perineum. Before pulling the testis into the pouch formed between the skin and dartos fascia of the left scrotum through a subcutaneous tunnel, the spermatic cord must be fully dissociated and the patent processus vaginalis needs to be transected and highly ligated. The left scrotum, testis, and spermatic cord were in a torsion-free and tension-free position (Figures 3A–D).

Healing well, the left scrotum was befitting to accommodate the left testis after the operation (Figure 1B). During the follow-up of 1 year, both the left and right scrotums and testes of this child developed normally. The parents were satisfied with the results of the surgery. They expressed their willingness to undergo another plastic surgery to achieve a more beautiful appearance in the future if possible.

## Discussion

There are four types of congenital scrotal anomalies that include penoscrotal transposition, bifid scrotum, ES, and accessory scrotum. The first two are more common, while the latter two are rare (12, 13). From the point of view of embryonic development, there is a hypothesis that ES is associated with defective gubernaculum formation during the conception period. Labioscrotal swellings and genital tubercle initiate the development of the scrotum and penis, respectively. Labioscrotal swellings move down and fuse to form the scrotum. The condensed mesenchymatous tissue develops into the gubernaculum, inserting into the labioscrotal swellings subsequently. At the same time, the testes descend into the scrotum. ES and concurrent ectopic testes are the results of defective gubernaculum formation, which can be caused by mechanical, genetic, chromosomal, or teratogenic damage. Rare ectopias can be caused by abnormal migration or premature fission of the labioscrotal swellings (2, 12, 14, 15).

Reviewing the literature, we found that most ES were associated with other congenital malformations, but a few of them complicated with VATER/VACTERL syndrome were reported. Spears et al. and Hasan et al. have reported two patients have ES with VATER association and ES with VACTERL association, respectively (14, 16). Some genitourinary abnormalities, such as cryptorchidism and renal hypoplasia, often occur on the ipsilateral side of the ES (3, 4, 12). However, we did not detect other significant abnormalities of the urinary system in the case we reported except for penoscrotal transposition. The child we reported did not have tracheoesophageal fistula with esophageal atresia, radial or renal dysplasia, limb anomaly, or other intracardiac malformations except PDA. Solomon emphasized that patent ductus arteriosus or patent foramen ovale should be considered normal age-based findings and not as components of the VATER/VACTERL association (7). Therefore, the diagnosis of VATER/VACTERL association was not appropriate in this case (6). Nonetheless, we recommend that it is needed to check whether there is a combination of VATER/VACTERL association or other syndromes if we meet newborns with ES although the probability is very low. According to Solomon et al. (5), the diagnosis of VATER/VACTERL association can be confirmed by examining each system separately. After confirming the presence of ARM during the physical examination, we can consider doing a prone cross-table lateral pelvic x-ray because it contributes to guiding the choice of surgery. Besides the clinical manifestations, the diagnosis of tracheoesophageal fistula can also be supported by radiological examinations, which indicate a coiled nasogastric/orogastric tube or an absent gastric bubble. Whether there are vertebral deformities or limb abnormalities can also be further examined by x-rays. In addition, ultrasonography can be routinely selected to rule out abnormalities in the heart or urinary system. Then, we can treat potential abnormalities and restore the physiological structure and function as far as possible.

As for ES, there are several operative methods for further discussion in the treatment. Although these papers provide various methods to treat ES, there is no clear guidance as to which operative method is superior. Hasan et al. (16) and Sharma et al. (17) chose to remove the ES and implant the ectopic testis into the normal scrotum. Similarly, Spears et al. (14) removed the ES and transferred a pedicled skin flap from the right scrotum to create a new left scrotum, fixing the left testis in the newly built scrotum. In addition, it was also feasible to establish a full-thickness perineal pedicled skin flap between the normal scrotum and the ES, which was transferred laterally and sutured to the ES (18). Daniel and Coleman (4) selected staged surgical management, which was composed of rotation flap scrotoplasty and orchidopexy. Orchidopexy was performed 6 months later. Zheng et al. performed orchiopexy and scrotoplasty for ectopic scrotum and released surrounding tissue of the inconspicuous penis (19). However, the most common operation is one-stage scrotoplasty with rotating skin flap and orchiopexy (1, 2, 12, 20). According to our own experience, the advantage of this operative method is that the donor vessels of the ectopic scrotal skin flap are preserved, which helps avoid tissue ischemic necrosis after the operation. More importantly, it is especially suitable for situations where the normal scrotum is not enough to accept both testes. However, it is feasible to remove the excess ES if the normal scrotum has enough space to accommodate two testes. In addition, we should also pay attention to the distinction between the accessory scrotum and ES before choosing the operative method. Often, there is a testis that is needed to be preserved on the ipsilateral side of ES. Unlike ES, the accessory scrotum without a testis can be simply removed (13). Therefore, it is particularly important to evaluate the existence of the ipsilateral testis before the operation. We suggest that the presence of testes and their blood supply should be determined by ultrasound, MRI, or CT. In the course of the operation, we can use laparoscopic exploration to find testes more pertinently. It is helpful to determine whether the deep inguinal rings are closed and whether the shapes of spermatic vessels and vas deferens are normal. It is reported that the appropriate time for scrotoplasty and orchiopexy for a child is when they are 6–12 months old (1). However, the child we reported underwent the operation at the age of 2 years and 5 months. The postoperative follow-up showed that the recovery was ideal. The development and blood circulation of the scrotum, testes, and epididymis are satisfactory. Additionally, we can correct the penoscrotal transposition and asymmetry of the bilateral scrotum at the second operation if the parents have a request.

## Data Availability

The original contributions presented in the study are included in the article/Supplementary Material, further inquiries can be directed to the corresponding authors.
